# A Multi-Teacher Knowledge Distillation Framework for Enhancing the Robustness of Automated Sperm Morphology Assessment

**DOI:** 10.3390/diagnostics16081230

**Published:** 2026-04-20

**Authors:** Osman Emre Tutay, Hamza Osman Ilhan, Hakkı Uzun, Merve Huner Yigit, Gorkem Serbes

**Affiliations:** 1 Department of Computer Engineering, Yildiz Technical University, Istanbul 34220, Türkiye; emre.tutay@std.yildiz.edu.tr (O.E.T.); hoilhan@yildiz.edu.tr (H.O.I.); 2 Department of Urology, Faculty of Medicine, Recep Tayyip Erdoğan University, Rize 53100, Türkiye; 3 Department of Biochemistry, Faculty of Medicine, Recep Tayyip Erdoğan University, Rize 53100, Türkiye; merve.huner@erdogan.edu.tr; 4 Department of Biomedical Engineering, Yildiz Technical University, Istanbul 34220, Türkiye; gserbes@yildiz.edu.tr

**Keywords:** knowledge distillation, multi teacher learning, sperm morphology classification, class imbalance, infertility

## Abstract

**Background/Objectives:** The manual analysis of sperm morphology, crucial for male infertility diagnosis, is subjective and time-consuming. Automated methods using deep learning, offer a promising alternative; however, standard deep models are prone to overfitting when applied to small, heavily unbalanced clinical datasets, limiting their generalization capability. This study proposes a knowledge distillation approach that functions as a strong regularizer, improving the robustness of automated sperm morphology analysis. **Methods:** We utilize soft distillation to transfer knowledge from a set of high-capacity teacher models to a smaller student model (SwinV2-base). The teacher architectures include SwinV2-large, EfficientNetV2-m, and ConvNeXtV2-large. To maximize performance, we investigated two distillation strategies: a single-teacher approach, where the student learns from one specific architecture, and a multi-teacher approach, where the student learns from an averaged response of multiple teachers. The models were trained on the imbalanced Hi-LabSpermMorpho dataset, which comprises 18 different sperm morphology categories derived from three differently stained (BesLab, Histoplus, GBL) sample sets. We adopted a cross-dataset training approach in which the teacher models were fine-tuned using the combination of two stained datasets, and the student model was trained on the third, distinct stained dataset. The global loss function combined cross-entropy loss with Kullback–Leibler divergence, employing the teacher’s soft probabilities to prevent the student from over-confidence. **Results:** The experimental results demonstrate that the student model trained in a multi-teacher setup with augmentation and soft distillation attains higher accuracies (70.94% on BesLab, 73.61% on Histoplus, 71.63% on GBL) than the baseline models. **Conclusions:** This approach mitigates challenges associated with data scarcity and heavily unbalanced sperm morphology datasets, providing consistent improvements and offering a highly generalizable solution for clinical diagnostics.

## 1. Introduction

The analysis of sperm morphology plays a critical role in the diagnosis of male infertility, a significant health concern affecting a substantial proportion of couples globally [1]. Traditionally, this analysis is performed manually by trained professionals using microscopy, a process recognized as subjective, time-intensive, and prone to inter- and intra-laboratory variability [2]. To address these limitations, there has been a growing need for automated alternatives, leveraging advancements in image analysis and machine learning.

Initial automated approaches relied on conventional computer vision and machine learning workflows, involving image preprocessing and sperm cell segmentation, followed by manual feature engineering and subsequent classification with traditional algorithms. For example, wavelet transform based noise reduction [3] and traditional image processing approach [4] based segmentation steps are frequently used in the literature to isolate individual sperm cells. However, these conventional techniques often struggle to distinguish overlapping structures in dense samples. Addressing this challenge, recent frameworks such as SpeHeaTal [5] employ cluster-enhanced deep learning to accurately segment complex, intersecting sperm tails. Furthermore, the focus has recently shifted toward multi-part semantic segmentation; for instance, Lei et al. [6] systematically demonstrated that while instance-based models like Mask R-CNN excel at delineating rigid structures (head and nucleus), U-Net architectures remain superior for capturing the fine, flexible details of the sperm tail. Various feature extraction approaches including the computation of shape and texture descriptors (e.g., head eccentricity, area, or intensity profiles) were applied to sperm images with the aim of capturing morphologic defects [7,8]. Feeding these features into classifiers (such as support vector machines or k-nearest neighbour) to categorize sperm as normal or abnormal was also investigated [9,10,11]. These traditional methods often rely on handcrafted feature design and expert tuning. Additionally, such approaches may consist of sequential sub-steps like feature selection and require domain-specific expertise. Moreover, they can struggle with variability in sperm appearance (e.g., overlaps, artifacts) and may lack robustness when applied to new datasets. Thus, while classical machine learning based approaches achieved useful results, they remained limited by the need for manual feature engineering and domain knowledge [12,13].

Image classification techniques are fundamental to automate the analysis of sperm morphology [14]. In recent years, deep learning – especially Convolutional Neural Networks (CNNs) – has transformed image-based diagnosis. Rather than handcrafting features, CNNs learn hierarchical features directly from raw pixels. CNNs integrate the entire analysis into an end-to-end pipeline. In practice, a CNN can directly process a sperm image, automatically learning filters to detect relevant morphological patterns by eliminating the need for manual pre-processing [15]. Yet, deploying such deep learning architectures entails substantial costs. Large CNNs typically have millions of parameters and require substantial computation, making them resource-hungry. In practical settings like fertility clinics, it may not be feasible to run very deep networks on-site due to limited hardware (e.g., only standard computers or embedded devices). This poses a key problem: achieving the predictive power of large CNNs while maintaining the efficiency and compactness needed for real-world deployment [16,17].

Knowledge Distillation (KD) offers a solution to this dilemma. Knowledge distillation has emerged as a promising machine learning technique to bridge the gap between the high accuracy achievable with complex deep learning models and the practical limitations of their deployment [18]. This technique involves transferring the knowledge from a large, well-performing model (the teacher) to a smaller, more efficient model (the student) without significant loss in performance [19]. The distillation process can be thought of as introducing a regularization effect: the large teacher captures rich data patterns (including class soft-probabilities and intermediate representations), and the student learns to reproduce those patterns. By first training a cumbersome model on possibly vast data and then distilling it into a smaller model, one can retain most of the teacher’s performance in a much more compact form. However, while knowledge distillation has been increasingly adopted in biomedical imaging, prior studies predominantly rely on single-teacher paradigms trained within a single data domain. These conventional approaches often struggle when applied to datasets characterized by severe class imbalance and high inter-domain variability (such as varying clinical staining protocols), as the student model tends to inherit the architectural biases and domain-specific overfitting of its teachers.

To address this methodological gap, this study proposes a dual-faceted innovation: a heterogeneous multi-teacher knowledge distillation framework combined with a cross-stain distillation protocol. First, our core architectural innovation lies in aggregating supervision from distinct, high-capacity teacher models—explicitly combining the global self-attention mechanisms of Vision Transformers (e.g., SwinV2-large) with the strong local inductive biases of modern Convolutional Neural Networks (e.g., ConvNeXtV2-large). Second, to ensure robust generalization across different clinical environments, we introduce a cross-dataset training strategy. By fine-tuning the teacher ensemble on a combination of different staining subsets (e.g., Histoplus and GBL) and distilling this aggregated, domain-enriched knowledge into a student model evaluating a completely distinct target stain (e.g., BesLab), our framework actively bridges the domain gap.

This dual approach mitigates the risk of architectural bias and prevents the minority class collapse that frequently plagues automated sperm morphology analysis. Our study aims to present a knowledge distillation approach to overcome certain challenges that occur when training an image classification model with a relatively small (unlike many computer vision tasks like ImageNet [20], COCO [21] and JFT [22]) and heavily unbalanced sperm morphology image dataset, namely Hi-LabSpermMorpho [23]. State-of-the-art ViT models need very large datasets to perform well. Without distillation, a large model (teacher) tends to overfit very fast on limited data. However, with knowledge distillation, large teacher models can be used to transfer knowledge and perform regularization to smaller student models with the aim of better generalization. In this way, our student model inherits the teacher’s learned structure and can perform reliably with far fewer parameters.

The rest of the paper is organized as follows: Section 2 reviews related applications of knowledge distillation and automated sperm analysis. Section 3 describes the materials and methods, including the dataset description and data acquisition approach, data pre-processing steps, knowledge distillation approach, deep learning models and performance metrics. The experimental results are discussed in Section 4, and finally, the significance of these results and the contributions of this study are addressed in Section 5.

## 2. Related Works

### 2.1. Traditional Approaches to Sperm Morphology Classification

In clinics, sperm morphology analysis involve manual examination under a microscope, a process known to be subjective, time-consuming, and susceptible to human error. To overcome these limitations, Computer-Aided Sperm Analysis (CASA) systems were developed to automate and standardize the evaluation of sperm parameters, including morphology [24]. These systems utilize digital image processing techniques to analyze sperm images and provide objective measurements and classifications [25].

The initial generation of these automated systems, emerging in the early 1990s, employed a “feature-engineering first” approach characterized by explicit geometric rules. A seminal validation of this methodology was performed by Kruger et al. [26] using the FERTECH system. This system relied on intensity thresholding for segmentation and calculated specific morphometric parameters—such as head length, width, ellipticity, and roughness—by measuring the major and minor axes of a best-fit ellipse. Classification was binary and deterministic: a sperm was deemed “Normal” only if all measured parameters fell within strict, pre-programmed ranges. While clinically prognostic, these systems functioned as rigid “black boxes” that struggled with borderline forms.

As computing power advanced, researchers transitioned from rigid heuristics to learning-based systems capable of handling biological non-linearity. Yi et al. [27] introduced the use of Artificial Neural Networks (ANNs), specifically Multi-Layer Perceptrons (MLP), to classify sperm heads. A key innovation in their work was the extraction of Invariant Fourier Descriptors (IFDs) and moment invariants, which allowed the classifier to recognize shapes regardless of rotation or orientation.

In recent years, there has been a growing trend towards employing deep learning approaches, particularly CNNs, for the task of sperm morphology classification [2,28,29,30,31,32]. These models have demonstrated the ability to automatically learn relevant features from sperm images and classify them into categories such as normal and abnormal based on the morphology of the head, midpiece, and tail. Some advanced deep learning models can even classify sperm into specific types of abnormalities, providing more detailed diagnostic information [23,33,34].

Several datasets have been specifically curated to facilitate research in automated sperm morphology analysis. These include Modified Human Sperm Morphology Analysis (MHSMA) dataset [35], Human Sperm Head Morphology (HuSHeM) dataset [33], Hi-LabSpermMorpho dataset [23] and SCIAN-MorphoSpermGS dataset [34]. Researchers have leveraged these benchmarks to introduce diverse deep learning methodologies. For instance, Shahzad et al. [1] utilized the MHSMA dataset to validate a sequential deep neural network capable of detecting abnormalities across the acrosome, head, and vacuole regions, achieving accuracies of 89%, 90%, and 92%, respectively. Addressing the geometric variability in the HuSHeM dataset, Guo et al. [2] proposed a framework integrating EdgeSAM with a pose correction network, significantly enhancing classification robustness against sperm orientation changes and achieving a test accuracy of 97.5%. While these datasets have been instrumental in advancing the field, they often present challenges such as low image resolution and clarity, the presence of noise and artifacts, data imbalance between normal and abnormal sperm, limited overall size, and inherent subjectivity in the manual labeling process. The performance of sperm morphology classification models is typically evaluated using metrics such as accuracy, precision, recall, F1-score, and the Area Under the Receiver Operating Characteristic curve (AUC-ROC). Despite progress, achieving reliable multi-label classification across all sperm parts remains challenging, motivating ongoing model and dataset improvements. To this end, recent studies have utilized the comprehensive Hi-LabSpermMorpho dataset to benchmark novel architectures. Turkoglu et al. [36] developed a category-aware two-stage divide-and-ensemble framework, achieving classification accuracies of 69.43%, 71.34%, and 68.41% across three staining protocols.

### 2.2. Knowledge Distillation for Sperm Morphology Image Classification

Knowledge distillation has proven to be a valuable technique in various image classification tasks, serving as an effective method for both compressing large, complex models and enhancing the performance of smaller ones [37]. By enabling the training of student models that can achieve accuracy levels comparable to their larger teacher counterparts, knowledge distillation facilitates the deployment of deep learning solutions in resource-constrained environments [18].

Several distinct strategies for knowledge distillation have been developed. Response-based distillation is a common approach where the student model is trained to directly mimic the output probabilities of the teacher model, often using soft targets. Feature-based distillation involves guiding the student’s learning by encouraging it to match the intermediate representations learned by the teacher, allowing for the transfer of richer semantic information. Relation-based distillation goes beyond individual layer outputs and focuses on transferring the relationships between different layers or data samples, capturing more holistic knowledge. Other notable strategies include adversarial distillation, which utilizes Generative Adversarial Networks (GANs) to improve the student’s ability to learn data distribution, and attention-based distillation, which transfers knowledge on the importance of different feature embeddings using attention mechanisms [16].

Research has highlighted the significant impact of the datasets used during training on the effectiveness of knowledge distillation. Characteristics of the datasets, such as their size, the specificity of the domain, and any inherent biases they might contain, can profoundly influence the knowledge transfer process [18].

The intersection of knowledge distillation and sperm morphology image classification has been explored in several recent studies. These investigations aim to leverage the benefits of knowledge distillation to improve the accuracy of sperm morphology classification models, address the challenges associated with limited and noisy datasets, and potentially create more efficient models suitable for clinical deployment.

A notable study [38] utilized the MHSMA dataset to train a model capable of distinguishing between normal and abnormal sperm cells. This research focused on a less-supervised approach, training an anomaly detection model using only normal sperm samples and employing knowledge distillation to enhance its performance despite the challenges of low-resolution and blurry images. The results demonstrated that this method could achieve Receiver Operating Characteristic (ROC)/Area Under the Curve (AUC) scores of 70.4%, 87.6%, and 71.1% for the head, vacuole, and acrosome, respectively. These figures indicate that the method can achieve performance comparable to traditional deep learning models while using significantly less data, suggesting its potential for deployment on edge devices in fertility clinics.

Another significant contribution is [14], which introduced a novel framework that leverages anatomical and image priors to perform unsupervised distillation of sperm head information using pseudo-masks and spatial prediction tasks. The framework achieved state-of-the-art results on two publicly available sperm morphology datasets, SCIAN and HuSHeM, attaining accuracies of 65.9% and 96.5%, respectively, effectively addressing the challenge of limited and potentially noisy labeled data. The potential benefits of applying knowledge distillation to sperm morphology image classification include improved accuracy, particularly when dealing with limited labeled data, and the possibility of creating more efficient models suitable for clinical use. However, a critical challenge remains regarding the domain gap; since standard teacher models are predominantly pretrained on ImageNet (natural images), they often struggle to fully adapt to the specific textural and structural characteristics of sperm morphology datasets without effective intervention. Furthermore, existing knowledge distillation methods are largely insufficient for heavily imbalanced and domain-variable biological datasets. In standard single-domain setups, models frequently develop a two-fold bias: they overfit to majority classes, and they memorize the specific visual artifacts of their training domain (e.g., variations in staining protocols). When this biased probability distribution is distilled, the student model inherits these vulnerabilities, leading to poor generalization on unseen clinical samples and a severe drop in sensitivity for underrepresented morphological defects.

The framework proposed in this study directly addresses these specific limitations. First, by aggregating the softened logits of architecturally distinct teacher models with equal weighting, the framework prevents the distillation process from disproportionately favoring any single architecture’s majority-class bias. Second, we introduce a cross-dataset training strategy to act as a rigorous domain-level regularizer. Because different clinical staining protocols (such as BesLab, Histoplus, and GBL) produce slightly different data distributions with distinct contrast and noise characteristics, fine-tuning the teacher ensemble on a mixture of these distributions and distilling into a student on a separate target stain forces the network to learn robust, stain-invariant morphological features rather than domain-specific artifacts. Together, these strategies force the student to learn from a more generalized, consensus-based representation, effectively mitigating both minority class collapse and cross-domain degradation in heavily skewed clinical data.

To explicitly position our contribution against existing literature, Table 1 summarizes the recent knowledge distillation approaches applied to sperm morphology. While previous methods [14,38] have successfully utilized distillation for binary classification or single-part anomaly detection (e.g., head only) within isolated datasets, they often struggle when subjected to extreme class imbalance and inter-domain clinical variations. Our proposed framework addresses these critical gaps by simultaneously classifying 18 fine-grained morphological defects across distinct staining protocols. By substituting the conventional single-teacher paradigm with a heterogeneous multi-teacher ensemble, our method actively mitigates architectural bias and minority class collapse, presenting a more comprehensive solution for real-world clinical deployment.

## 3. Materials and Methods

### 3.1. Dataset Information and Preprocessing

**Figure 1:**
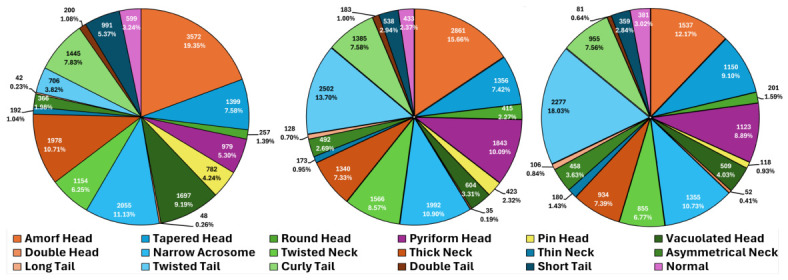
Distribution of classes in dataset according to BesLab (**left**), Histoplus (**middle**) and GBL (**right**).

The Hi-LabSpermMorpho datasets consist of sperm images prepared with three different Diff-Quick stains (BesLab, Histoplus, and GBL) [23]. All three subset share an identical label space of 18 detailed morphology classes (including a normal class) spanning head, midpiece (neck), and tail abnormalities. In practice, each spermatozoon was first cropped from the raw microscope images using coordinates provided by a single expert. Subsequently, two experts evaluated these cropped images and assigned the final morphological labels through a joint consensus, following WHO sperm morphology criteria [39]. This dual-expert, WHO-standard annotation procedure yields a high-quality ground truth. As it is a typical problem in sperm morphology datasets, the class distribution is highly skewed: for example, amorphous head, thick/twisted neck, and twisted tail defects are very common, while other classes (e.g., “Long Tail”) have only a few dozen examples [23]. To train and validate our model, we performed 5-fold cross-validation (each fold with an 80% train/20% test split). The distribution of classes in the dataset can be seen on Figure 1 and Table 2.

Any potential data leakage during cross-dataset training is inherently prevented by the structural design of the dataset itself. The data collection process is strictly isolated at the patient level; each patient underwent a separate, independent acquisition session, and their resulting morphological samples belong exclusively to a single staining subset (BesLab, Histoplus, or GBL). Because of this inherent organization, the subsets are entirely independent, with absolutely no patient overlap or shared acquisition sessions across the different staining protocols. Each of the three subsets of Hi-LabSpermMorpho datasets differs only by its staining protocol. These clinical staining variations lead to distinct image characteristics. For instance, BesLab-stained slides (the standard clinic kit) tend to show smoother transitions and less noise—although some fine structures may be lightly stained or missed. The GBL stain produces higher contrast and heavier dye uptake on the sperm heads. Histoplus preparations often exhibit dense background sedimentation and pronounced tail details (especially in high-sperm-count samples), at the expense of more background artifacts [23]. By including all three, we ensure our model is evaluated under realistic variations in staining and imaging conditions.

Prior to modeling, we applied standard preprocessing and augmentation. First, each raw sperm image was down-scaled to 256 × 256 to normalize the field of view. We then used PyTorch (version 2.10.0, Meta Platforms, Inc., Menlo Park, CA, USA) and its torchvision (version 0.25.0) [40] transforms to expand the dataset to six times its original size (the original image plus five augmented versions). The augmentation strategies were designed to introduce realistic geometric variation while preserving morphology. After the pre-processing steps, five distinct transformation strategies were applied exclusively to the images in the training set; these, combined with the original training images, increased the training data size by a factor of six. Transformations were carefully selected to introduce meaningful variations while preserving essential image characteristics. Specifically, the biological nature of the sperm morphology task heavily constrained the acceptable augmentation parameters:

- Rotation ($\pm 30^{\circ }$): Selected to introduce orientation variance, mimicking the natural angular variations of sperm cells on a slide. Larger rotations were avoided to prevent the elongated tail structures from being cut off by the 256×256 crop boundaries.
- Translation (Width and Height Shift 0–0.1): Minor spatial shifts were incorporated to compensate for potential slight misalignments or off-center positioning that occurred during the manual, expert-guided bounding box cropping phase.
- Horizontal Flipping: Applied with a probability of p=0.5 to enhance viewpoint diversity, reflecting the arbitrary left-right positioning of the cells under the microscope.
- Shear ($10^{\circ }$) and Zoom (0.9–1.0): These subtle operations were utilized to simulate the physical, fluid-induced bending of the sperm tail and slight variations in focal magnification, respectively.

**Figure 2:**
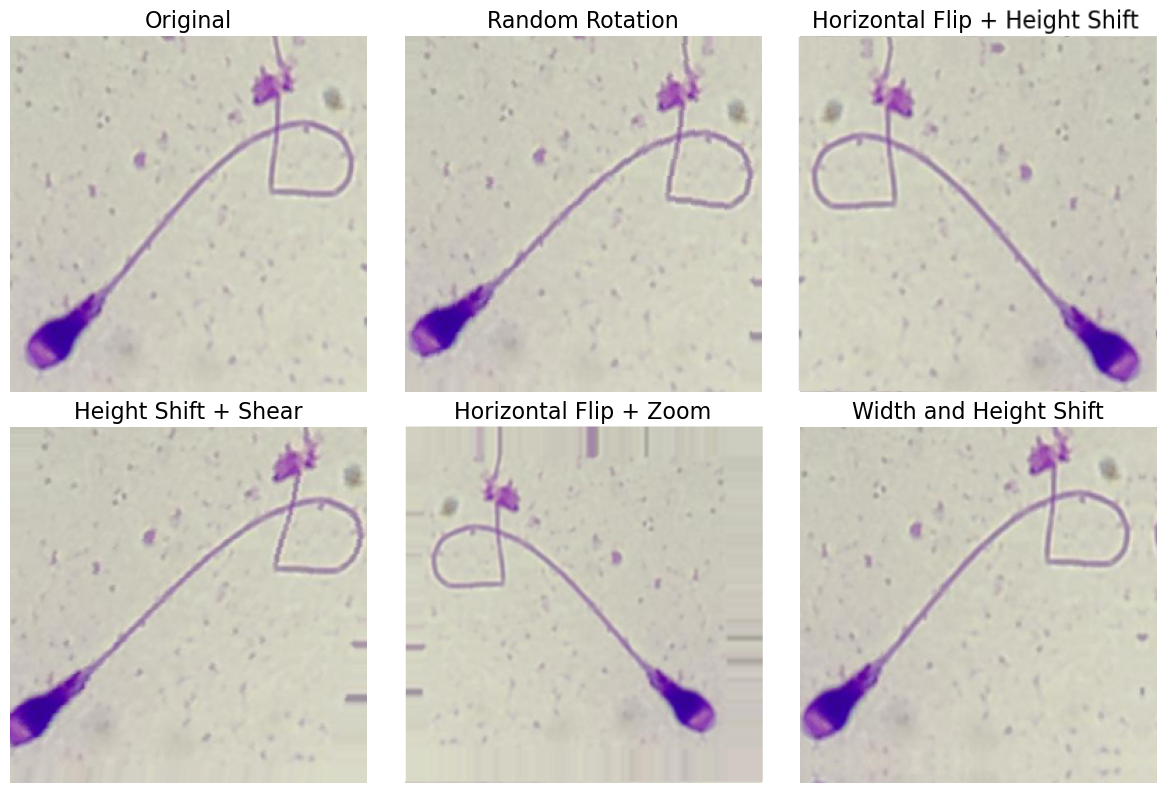
Example of the original image alongside five applied data augmentation strategies.

Crucially, more aggressive distortions were deliberately excluded to maintain the structural integrity required to successfully distinguish between highly similar morphological classes (e.g., distinguishing a ‘Thick Neck’ from a ‘Normal’ neck). The details of the implemented augmentation strategies are also listed in Table 3, and visual examples of these transformations are shown in Figure 2. Each transform was applied after appropriate padding (mode and size chosen to avoid artifacts), followed by a central 256 × 256 crop to maintain input size. This augmentation markedly increases training diversity and helps mitigate overfitting.

To mitigate overfitting and improve the robustness of the feature representations, we integrated Stochastic Depth (SD) and Label Smoothing (LS) into our training pipeline. SD treats the depth of the network as a random variable. During training, for a residual block *l*, a Bernoulli random variable $b_{l}\in \left\{0,1\right\}$ determines whether the block is active ($b_{l}=1$) or inactive ($b_{l}=0$). Formally, the output of the *l*-th layer $H_{l}$ is given by:

(1) $$H_{l}=H_{l-1}+b_{l}f_{l}\left(H_{l-1}\right)$$

where $f_{l}$ represents the transformation of the residual block (typically incorporating Layer Normalization, Self-Attention/MLP, and GELU activation) and $H_{l-1}$ represents the identity mapping of the input. During inference, the full network is used, and the weights are typically scaled by the survival probability $p_{l}$.

Standard cross-entropy loss often leads to overconfident predictions on the training set. We replaced the one-hot encoded hard targets *y* with smoothed targets $y^{LS}$ in LS. For a class *k* and a total of *K* classes, the smoothed label is calculated as:

(2) $$y_{k}^{LS}=\left(1-\epsilon \right)y_{k}+\frac{\epsilon }{K}$$

where ϵ is a small smoothing parameter (0.1). This approach penalizes low entropy output distributions, ensuring better generalization on unseen data.

### 3.2. The Implementation Details of Knowledge Distillation

Knowledge distillation is a model compression technique that transfers knowledge from a large, complex model (teacher) to a smaller, more efficient model (student). In this study, soft distillation was used. Soft distillation represents a specialized form of knowledge distillation that focuses on transferring knowledge through probability distributions rather than hard class labels. Soft distillation minimizes the Kullback–Leibler divergence between the softmax of the teacher and the softmax of the student model. Let $Z_{t}$ be the logits of the teacher model, $Z_{s}$ the logits of the student model. We denote by τ the temperature for the distillation, λ the distillation weight balancing the Kullback–Leibler divergence loss (KL) and the cross-entropy ($L_{\mathrm{CE}}$) on ground truth labels *y*, and ψ the softmax function. The global loss function for distillation is given by:

(3) $$L_{\mathrm{global}}=\left(1-\lambda \right)L_{\mathrm{CE}}\left(\psi \left(Z_{s}\right),y\right)+\lambda \tau^{2}\mathrm{KL}\left(\psi \left(Z_{s}/\tau \right),\psi \left(Z_{t}/\tau \right)\right).$$

As shown in Equation (3), the global loss combines both the cross-entropy loss and the Kullback–Leibler divergence between the teacher and student models.

**Figure 3:**
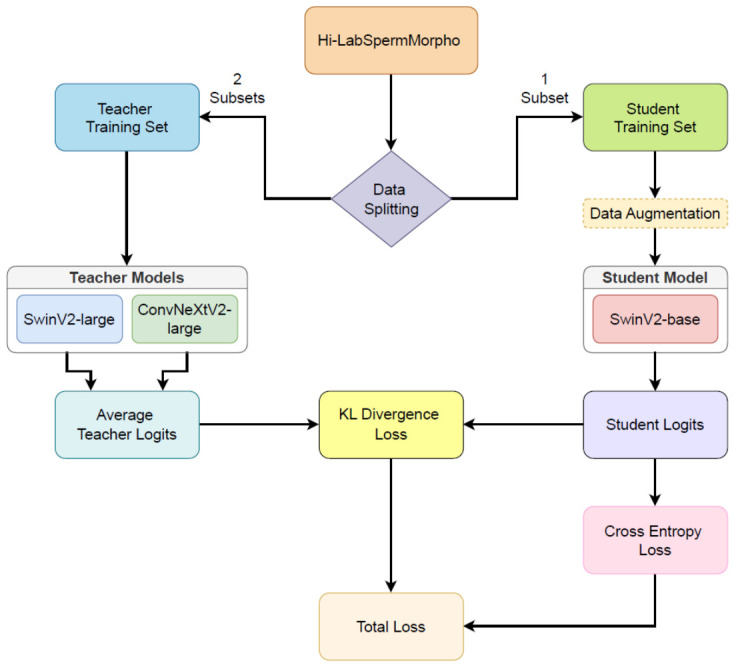
Flowchart of the system.

In the proposed system, soft distillation was used to train the classification model for mitigating the effects of overfitting. Finally, the obtained model is evaluated by the performance metrics, and these metrics are reported. The flow chart of the study is demonstrated in Figure 3. To enhance robustness, a multi-teacher framework was employed in which the SwinV2-base student model learns from an ensemble of high-capacity teacher architectures, specifically SwinV2-large and ConvNeXtV2-large. Both models pre-trained on ImageNet-21k and fine-tuned on ImageNet-1k datasets. To ensure strict reproducibility, the exact pre-trained weights for the architectures were sourced from publicly available libraries prior to our domain-specific fine-tuning. The SwinV2 and ConvNeXtV2 architectures were initialized using the Hugging Face transformers library (version 5.0.0, Hugging Face Inc., Brooklyn, NY, USA), utilizing the specific checkpoint identifiers microsoft/swinv2-base-patch4-window12to16-192to256-22kto1k-ft for the student model, and microsoft/swinv2-large-patch4-window12to16-192to256-22kto1k-ft alongside facebook/convnextv2-large-22k-224 for the teacher ensemble. Additionally, where applicable, the EfficientNetV2-M weights were sourced directly from the PyTorch torchvision.models library. Two subsets of the Hi-LabSpermMorpho datasets were combined and used to fine-tune the teacher models, while the remaining subset was utilized in the student model training and performance evaluation steps. To ensure comprehensive evaluation across all domains, this cross-dataset training strategy was systematically repeated for all dataset combinations using a leave-one-out protocol. Specifically, this rotation resulted in three distinct training cycles: (1) the student model was trained on BesLab while teachers were fine-tuned on Histoplus and GBL; (2) the student was trained on Histoplus while teachers were fine-tuned on BesLab and GBL; and (3) the student was trained on GBL while teachers were fine-tuned on BesLab and Histoplus. The training parameters for both the student and teacher models are detailed in Table 4.

In the training of student model, we utilized the AdamW optimizer with a learning rate of 0.00002, chosen for its efficient convergence in optimization tasks and its ability to handle weight decay effectively, which is essential in preventing overfitting during training. To further enhance the training process, we incorporated a learning rate scheduler, specifically the StepLR scheduler. This scheduler was configured with a gamma value of 0.45, meaning that the learning rate is reduced by a factor of 0.45 for first 5 epochs. This adjustment ensures that the model can stabilize and refine its learning in later stages of training, promoting convergence to a more optimal solution. The training was carried out over 10 epochs, which allowed sufficient iterations for the model to learn meaningful patterns from the data while preventing overtraining. Additionally, the batch size was set to 4, which was found to be a good balance between memory consumption and model performance. This batch size allows for efficient computation and model updates while avoiding excessive variance in gradient estimation. The optimal distillation weight was found to be 0.3, as the teacher model was trained on a data set that differs from the data set of the student model, leading to a trade-off between preserving the teacher’s knowledge and allowing the student to learn from its own data distribution. To allow the student to learn on its own, cross entropy loss was given more weight in the global loss. Due to the domain mismatch between the student and teacher models, τ was set to 8 to soften the predicted probability distributions and provide regularization. Stochastic depth of the student model was set to 0.3 to improve generalization abilities of the system. Also, label smoothing with value 0.1 used to make the model less overconfident. Due to the substantial computational cost of training high-capacity ensembles (SwinV2-large and ConvNeXtV2-large), implementing a formalized hyperparameter optimization algorithm (e.g., grid or random search) was computationally prohibitive. Consequently, hyperparameters were established through heuristic selection rather than exhaustive tuning. Standard architectural regularizers, such as Stochastic Depth (0.3) and Label Smoothing (0.1), along with the AdamW optimizer baseline, were adopted directly from established best practices in Vision Transformer literature to ensure a stable optimization landscape. The distillation-specific parameters, specifically the temperature (τ=8) and distillation weight (0.3), were selected heuristically during informal preliminary trials. These values were chosen to provide sufficient smoothing of the teacher logits, compensating for the domain mismatch between the teachers’ natural image pretraining and the target sperm morphology dataset. These hyperparameters were selected through experimentation and prior knowledge to ensure a robust model training process, optimizing for both generalization and computational efficiency.

The teacher models, which were fine-tuned on the aggregation of the remaining subsets, shared the same optimizer (AdamW), learning rate (0.00002), and batch size (4) as the student to ensure consistency in the optimization landscape. However, distinct adjustments were made to accommodate the fine-tuning process. The training duration was reduced to 4 epochs, which is sufficient for adapting pre-trained weights to the new domain without destroying learned features. To mitigate the risk of overfitting on the combined datasets, a significantly higher weight decay of 0.01 was applied. Furthermore, the learning rate scheduler was adjusted to a step size of 2 with a gamma of 0.5, decaying the learning rate more aggressively to settle the model weights into an optimal local minimum efficiently.

Although the training durations (10 epochs for the student and 4 for the teachers) may appear unusually short compared to training from scratch, these specific schedules were deliberately empirically determined as early-stopping mechanisms to prevent severe overfitting. Because the high-capacity architectures were initialized with robust pre-trained ImageNet weights, they converged very rapidly on the relatively small Hi-LabSpermMorpho dataset. Extended training beyond these epochs consistently led to model degradation and memorization of the majority classes due to the extreme class imbalance.

### 3.3. Multi-Teacher Knowledge Distillation Framework

To further enhance the generalization capability of the system and mitigate the risk of overfitting to specific architectural biases, we extended the single-teacher approach to a multi-teacher knowledge distillation framework. While the single-teacher baseline relies solely on the SwinV2-large architecture, the multi-teacher strategy incorporates additional high-capacity models to provide a more diverse set of feature representations. Specifically, we utilized EfficientNetV2-m [41] and ConvNeXtV2-large [42] as additional teacher architectures alongside SwinV2-large. EfficientNetV2-m was selected due to its status as the top-performing model on the Hi-LabSpermMorpho dataset, and ConvNeXtV2-large, chosen as it is established in recent comprehensive reviews as a highly robust backbone for medical image classification, consistently outperforming traditional CNNs and offering competitive accuracy to Transformers [43].

In this framework, the student model learns from the aggregated knowledge of the teacher ensemble as shown in Figure 3. Instead of mimicking the probability distribution of a single teacher, the student is guided by the averaged response of multiple teachers. Let *N* be the number of teacher models, and $Z_{t}^{\left(k\right)}$ be the logits produced by the *k*-th teacher. The effective teacher logits, ${\bar{Z}}_{t}$, used in the distillation process are calculated as the arithmetic mean of the individual teacher outputs:

(4) $${\bar{Z}}_{t}=\frac{1}{N}\sum_{k=1}^{N}Z_{t}^{\left(k\right)}$$

Consequently, in the global loss function (Equation (4)), the single teacher terms are replaced by this ensemble average. The loss calculation uses the softmax of these averaged logits, $\psi ({\bar{Z}}_{t}/\tau )$, to compute the Kullback-Leibler divergence against the student’s softened output. We investigated two specific multi-teacher configurations: pairing SwinV2-large with EfficientNetV2-m, and pairing SwinV2-large with ConvNeXtV2-large, to leverage the complementary strengths of Transformer-based and CNN-based architectures. Simple arithmetic averaging was deliberately chosen over performance-based weighting to act as a robust regularizer. In the context of heavily imbalanced data, equal weighting prevents the ensemble from disproportionately favoring a teacher that might overfit the majority classes, ensuring that the distinct inductive biases of both architectures contribute equally to the student.

### 3.4. Evaluation Metrics

Accuracy, precision, recall and F1-score metrics were used in this study. These metrics were calculated from confusion matrices according to true positives, true negatives, false positives, and false negatives. In the equations, TP (true positive) and TN (true negative) represent correctly classified positive and negative samples, respectively. Conversely, FP (false positive) and FN (false negative) denote misclassified samples, where FP refers to negative samples incorrectly identified as positive, and FN refers to positive samples incorrectly classified as negative. Accuracy is the most commonly used metric for evaluating model performance, it directly indicates the proportion of correctly classified samples. The precision score measures the accuracy of positive predictions by assessing how many of the predicted positive samples are correct. The recall metric indicates the percentage of actual positive samples that the system correctly identifies. The F1-score provides a more informative comparison by incorporating both precision and recall. Instead of using the arithmetic mean, it is calculated as the harmonic mean of precision and recall, as shown in Equation (5). The harmonic mean is preferred because it reduces the influence of outliers, ensuring a more balanced evaluation. The formula for the performance metrics is given in the following equations.

(5) $$\mathrm{F1-score}=\frac{2\times \mathrm{Precision}\times \mathrm{Recall}}{\mathrm{Precision}+\mathrm{Recall}}$$

To rigorously characterize the model’s behavior under severe class imbalance, we computed and reported macro-averaged and weighted-averaged precision, recall, and F1-score. In addition, to more comprehensively assess performance in this imbalanced setting and to better align with clinical safety requirements, we incorporated the Precision–Recall Area Under the Curve (PR-AUC). In contrast to overall accuracy or ROC curves, PR-AUC is highly sensitive to the performance on minority classes, making it particularly critical for the identification of rare morphological abnormalities, for which missed detections may have substantial clinical consequences. For the multi-class setting, PR-AUC is quantified as the Average Precision (AP) computed for each class across all decision thresholds, as defined in Equation (6).

(6) $$\mathrm{PR-AUC}=\sum_{n}\left(R_{n}-R_{n-1}\right)P_{n}$$

where $P_{n}$ and $R_{n}$ denote the precision and recall at the *n*-th threshold, respectively. The macro-averaged PR-AUC is then calculated by taking the unweighted mean of the per-class PR-AUC scores to transparently reflect the framework’s diagnostic capability across both frequent and rare morphological categories.

## 4. Results

The results of the first stage of the experiments, conducted exclusively on the BesLab subset, are summarized in Table 5. Seven different training settings were evaluated to assess the incremental impact of each component: the baseline model trained without augmentation or distillation (Exp. 1), the model trained with data augmentation only (Exp. 2), and the student model trained using soft distillation with SwinV2-large serving as the teacher (Exp. 3). To further enhance the generalization capability of the model, we incorporated two advanced regularization strategies. First, we employed Stochastic Depth, which randomly drops a subset of layers during training and bypasses them with the identity function. This technique effectively reduces the training time and acts as a regularizer by training an ensemble of shallower networks implicitly. Second, Label Smoothing was applied to prevent the model from predicting the training examples with overconfidence. By softening the “hard” ground truth labels with a uniform distribution, label smoothing encourages the model to learn more distinct and clustered representations, thereby mitigating overfitting. Consequently, to empirically validate these contributions, we further analyzed the impact of regularization by adding stochastic depth (Exp. 4) and label smoothing (Exp. 5) individually, followed by a configuration combining both techniques (Exp. 6). The final experiment (Exp. 7) utilizes a single teacher framework, where the SwinV2-large teacher was fine-tuned using the combined Histoplus and GBL datasets. Each model was evaluated using accuracy, weighted precision, weighted recall, and weighted F1-score metrics computed from the test set across all five folds.

To validate the performance gains observed in the single-teacher configurations, a paired t-test was conducted across the 5-fold cross-validation results on the BesLab dataset. As detailed in Table 6, the evaluation compared the Macro F1-score of the optimal single-teacher setup (Exp. 7) against the other six configurations. The statistical analysis confirms that the fully configured model (Exp. 7) achieves a statistically significant improvement (p<0.05) over the baseline model (Exp. 1, p=0.0334). Furthermore, it significantly outperforms intermediate distillation setups, including the baseline student-teacher model (Exp. 3, p=0.0134) and configurations incorporating stochastic depth (Exp. 4, p=0.0172; Exp. 6, p=0.0350). While the improvements over the augmentation-only model (Exp. 2, p=0.4223) and the label-smoothed distillation model (Exp. 5, p=0.0911) did not reach statistical significance, the overall absolute gains demonstrate that combining domain-adapted teacher training with a robust augmentation and regularization pipeline yields the most stable and performant feature representations.

**Figure 4:**
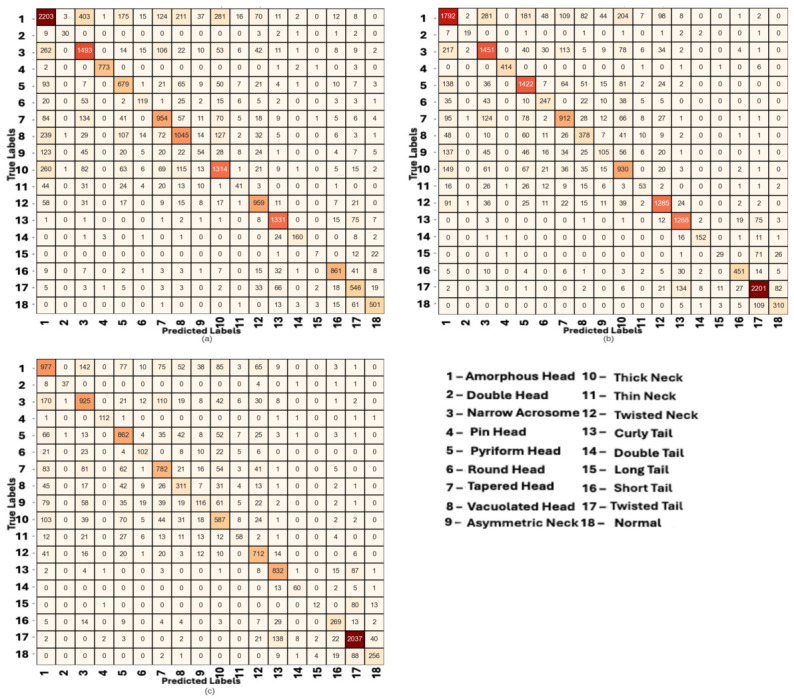
Confusion matrix of experiment settings 7 for single teacher distillation method (Teacher = SwinV2-large, Student = SwinV2-base) (**a**) Beslab, (**b**) Histoplus, (**c**) GBL. The color intensity in the confusion matrices represents the number of samples, with darker shades indicating higher frequencies.

Figure 4 illustrates the confusion matrices obtained from the best-performing single-teacher configuration (Experiment 7) across the BesLab, Histoplus, and GBL datasets. A visual inspection of the matrices reveals a strong diagonal dominance, indicating that the model correctly classifies the majority of samples despite the significant class imbalance. However, distinct patterns emerge when analyzing specific morphological subgroups. The model demonstrates high proficiency in identifying dominant classes such as ‘Amorphous Head’ (Class 1) and ‘Twisted Tail’ (Class 17). Interestingly, even some minority classes like ‘Double Head’ (Class 2) are classified with high precision, likely due to their distinctive visual features. Conversely, classes with subtle structural ambiguities, such as ‘Long Tail’ (Class 15) or ‘Thin Neck’ (Class 11), show relatively higher confusion rates. For instance, in the BesLab dataset (Figure 4a), misclassifications are frequently observed among neck and head abnormalities, suggesting that the model occasionally struggles to distinguish between fine-grained variations in these regions. Furthermore, we observed an interesting pattern regarding tail defects across the different staining protocols. In the Histoplus (Figure 4b) and GBL (Figure 4c) datasets, the correct prediction counts for tail defects (specifically Class 17) are notably higher compared to BesLab. While speculative without controlled, cross-stain optical experiments on identical samples, we hypothesize that this pattern may align with the staining characteristics described in Section 3.1. The Histoplus and GBL protocols often provide higher contrast or darker dye uptake on fine structures, which might make tail anomalies more discernible to the feature extractors compared to the smoother BesLab stain.

Table 7 presents a comprehensive comparison between the Single-Teacher baseline and the proposed Multi-Teacher distillation frameworks across all three datasets. When evaluating the impact of the Single vs. Multi-Teacher paradigms, the results indicate that aggregating knowledge from multiple teacher architectures generally yields a more robust student model. While the Single-Teacher (SwinV2-large) baseline already achieves competitive performance, the Multi-Teacher approach—particularly the configuration pairing SwinV2-large with ConvNeXtV2-large—consistently surpasses the baseline in F1-scores across all datasets (0.7052 for BesLab, 0.7324 for Histoplus, and 0.7107 for GBL). Regarding the teacher combinations, the synergy between the Swin Transformer and ConvNeXtV2 appears superior to the Swin and EfficientNetV2 pair. The SwinV2-large + ConvNeXtV2-large configuration achieved the highest metrics in nearly all categories. This can be attributed to the complementary nature of the teachers: SwinV2 excels in capturing long-range global dependencies via self-attention, while ConvNeXtV2 provides strong inductive biases for local feature extraction. This diversity seemingly offers a richer supervision signal to the student compared to the EfficientNet combination.

In terms of staining protocols, the Histoplus dataset consistently yielded the highest performance metrics (Accuracy: 0.7361 with Multi-Teacher), reinforcing the observation that its high-contrast characteristics facilitate easier feature discrimination. BesLab, with its smoother texture, remained the most challenging domain. Finally, analyzing the metrics, it is crucial to highlight the behavior on the GBL dataset. Although the Single-Teacher model achieved a marginally higher accuracy (0.7163) compared to the Multi-Teacher ConvNeXt configuration (0.7163), the latter achieved a higher Weighted F1-score (0.7107 vs. 0.7095). Since F1-score is the harmonic mean of precision and recall and is more sensitive to class imbalance, this improvement indicates that the Multi-Teacher framework provides better generalization for minority classes, preventing the model from merely overfitting to the majority class to maximize raw accuracy.

To rigorously verify the diagnostic advantages of the ensemble approach, Table 8 presents the results of a paired t-test conducted on the 5-fold cross-validation Macro F1-scores, comparing the Single-Teacher baseline (SwinV2-large) against the optimal Multi-Teacher framework (SwinV2-large + ConvNeXtV2-large). The statistical analysis confirms that the performance improvement, while appearing modest in terms of raw global accuracy, is statistically significant across all three staining protocols (p=0.0079 for BesLab, p=0.0464 for Histoplus, and p=0.0324 for GBL). Because the Macro F1 metric assigns equal weight to all classes regardless of their support size, this statistically significant gain mathematically validates that the heterogeneous Multi-Teacher framework systematically enhances the student model’s sensitivity to underrepresented morphological anomalies, proving that the improvement is a structural benefit of the distillation process rather than an artifact of random variance.

**Figure 5:**
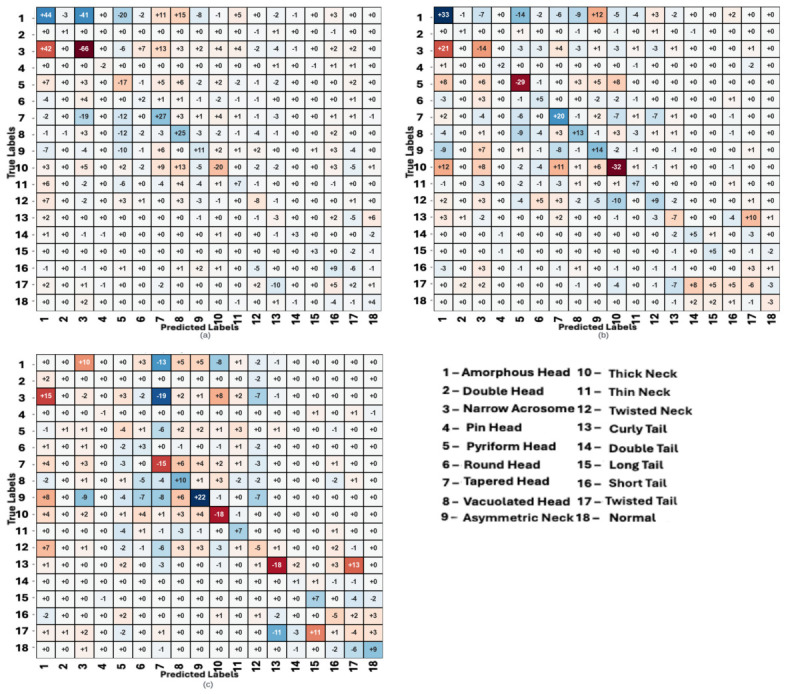
Difference Matrix between Multi Teacher and Single Teacher (Exp. 7) Distillation Approaches (Multi-Teacher = SwinV2-large and ConvNeXtV2-large, Student = SwinV2-base) (**a**) Beslab, (**b**) Histoplus, (**c**) GBL. Red colors represent positive differences, while blue colors represent negative differences.

Figure 5 visualizes the difference matrices calculated by subtracting the Single-Teacher confusion matrix from the Multi-Teacher confusion matrix ($M_{Multi}-M_{Single}$). In these heatmaps, blue tones indicate improvements (an increase in True Positives on the diagonal or a decrease in errors on off-diagonal cells), while red tones indicate performance deterioration. A granular analysis of these matrices reveals that the Multi-Teacher framework induces a significant shift in decision boundaries, characterized by a reduction in False Positives for the majority class at the expense of sensitivity in specific minority groups:

- Majority Class Bias and the Class 1–3 Trade-off: In the BesLab dataset (Figure 5a), a critical interaction is observed between ‘Amorphous Head’ (Class 1) and ‘Narrow Acrosome’ (Class 3). The Multi-Teacher model achieves a substantial improvement for Class 1 (Diagonal: +44), significantly reducing instances where Amorphous heads were mistaken for Narrow Acrosome (Row 1, Column 3: −41). However, this improvement comes with a penalty: the model becomes overly conservative regarding Class 3, leading to a sharp drop in its diagonal accuracy (−66) and a corresponding surge in misclassifying actual Narrow Acrosomes as Amorphous (Row 3, Column 1: +42). This suggests that the Multi-Teacher ensemble effectively minimizes false positives for the majority class but tends to absorb morphologically similar minority samples into the majority category.
- Consistent Robustness in Vacuolated Head (Class 8) and Asymmetric Neck (Class 9): Unlike the volatile classes, specific morphological defects show stable and significant improvements across all datasets. ‘Vacuolated Head’ (Class 8) exhibits remarkable diagonal gains, particularly in BesLab (+25), followed by Histoplus (+13) and GBL (+10). Similarly, ‘Asymmetric Neck’ (Class 9) shows consistent diagonal increases of +11 (BesLab), +14 (Histoplus), and +22 (GBL). This indicates that the Multi-Teacher architecture extracts more distinctive features for these specific anomalies, successfully distinguishing them from similar defects such as ‘Pyriform Head’ (Class 5), where confusion with Class 8 was reduced by 12 samples in the BesLab dataset.
- Systematic Sensitivity Struggles in Thick Neck (Class 10): Conversely, for ‘Thick Neck’ (Class 10), the Multi-Teacher model exhibits a consistent performance deterioration across all datasets: BesLab (−20), Histoplus (−32), and GBL (−18). This widespread drop in true positives, accompanied by increased confusion dispersed among various classes, suggests a systematic challenge. While we cannot definitively isolate the cause without further ablation studies, we hypothesize that the specific inductive bias of the Single-Teacher model may have been more advantageous for defining neck thickness. It is possible that the ensemble averaging process softened the sharp decision boundaries required to distinguish this specific midpiece defect from normal morphology.

In summary, the detailed confusion matrix analysis reveals a fundamental trade-off inherent in the multi-teacher distillation framework. By aggregating diverse architectural features, the ensemble significantly enhances prediction stability and minimizes false positives for overwhelmingly dominant majority classes (e.g., ‘Amorphous Head’). It also successfully captures distinctive features for certain underrepresented minority classes (e.g., ‘Asymmetric Neck’). However, this aggregated knowledge can inadvertently smooth out the sharp decision boundaries needed for morphologically ambiguous categories, occasionally reducing sensitivity for specific mid-sized or subtle classes (e.g., ‘Thick Neck’ or ‘Narrow Acrosome’). Ultimately, while the multi-teacher approach maximizes overall diagnostic robustness and broad anomaly detection, it highlights the ongoing challenge of preserving high sensitivity across all structurally similar minority classes in heavily imbalanced clinical datasets.

Table 9 details of the obtained class-wise performance across all three datasets. A closer inspection reveals that performance is highly correlated with the visual distinctiveness of the morphological defects. Classes with gross structural anomalies achieved exceptional results regardless of the staining technique. For instance, ‘Pin Head’ (Class 4), characterized by a significantly reduced head size, achieved near-perfect F1-scores of 0.9910, 0.9905, and 0.9573 for BesLab, Histoplus, and GBL, respectively. Similarly, ‘Double Tail’ (Class 14) and ‘Curly Tail’ (Class 13) consistently maintained F1-scores above 0.80, confirming that the model effectively captures large-scale geometric deformations. Furthermore, the inclusion of PR-AUC scores in Table 9 provides a stringent evaluation of the model’s reliability on minority classes. While macro-averaged F1-scores highlight overall balance, the per-class PR-AUC values explicitly confirm that the Multi-Teacher framework maintains high precision without sacrificing recall, even for heavily underrepresented morphologies like ‘Long Tail’ and ‘Double Head’.

The impact of the staining protocol, previously observed in the confusion matrices, is quantitatively confirmed here—particularly for tail defects. The ‘Twisted Tail’ (Class 17) category shows a marked performance jump in the Histoplus (F1: 0.8782) and GBL (F1: 0.8851) datasets compared to BesLab (F1: 0.7211). This substantial increase supports the premise that the higher contrast and dye uptake in Histoplus and GBL stains make the fine texture of twisted tails more discernible to the feature extractors. Conversely, the models struggled with minority classes exhibiting subtle midpiece or tail variations. ‘Asymmetric Neck’ (Class 9) and ‘Long Tail’ (Class 15) yielded the lowest F1-scores across the board (e.g., roughly 0.24–0.33 for Asymmetric Neck). The discrepancy between precision and recall in these classes is notable; for instance, in BesLab, ‘Asymmetric Neck’ has a precision of 0.4088 but a Recall of only 0.1776. This indicates that while the model is relatively conservative in predicting these labels, it misses a significant portion of true positive cases, likely due to the scarcity of training samples and the high inter-class similarity with ‘Asymmetric Neck’ or ‘Normal’ midpieces. Interestingly, the ‘Normal’ class (Class 18) achieved its highest performance in the BesLab dataset (F1: 0.8545), outperforming Histoplus (0.7173) and GBL (0.7378). This suggests that while high-contrast stains (Histoplus/GBL) excel at highlighting specific defects, they may also introduce artifacts that complicate the identification of perfectly normal morphology, whereas the smoother BesLab stain offers a clearer baseline for normality.

To provide a holistic view of the model’s diagnostic reliability, particularly concerning clinical safety and the detection of rare abnormalities, we evaluated the macro-averaged and weighted-averaged PR-AUC scores (Table 9). The proposed Multi-Teacher framework achieved robust weighted PR-AUC scores of 0.7673, 0.7965, and 0.7706 on the BesLab, Histoplus, and GBL datasets, respectively. These high weighted averages confirm the model’s strong overall predictive power across the heavily populated classes. More importantly, the macro-averaged PR-AUC scores remained highly competitive (0.7276 for BesLab, 0.7516 for Histoplus, and 0.7300 for GBL). Because the macro-average treats all 18 classes equally regardless of their support size, the relatively narrow gap between the weighted and macro averages mathematically demonstrates that our distillation strategy effectively mitigates minority class collapse. This ensures that rare but clinically significant morphological defects are not masked by the extreme class imbalance, thereby aligning the model’s performance with critical clinical safety standards.

Finally, to evaluate the computational efficiency gained through the proposed knowledge distillation framework, Table 10 presents a comparison of the model parameters and inference times. These inference measurements were obtained by processing one test image of the BesLab dataset using an NVIDIA RTX 4070 GPU (NVIDIA Corporation, Santa Clara, CA, USA). The student model (Swin V2-base) significantly reduces the computational burden, utilizing only 88.0 M parameters with an inference time of 6.64 ms. In contrast, the high-capacity teacher models, Swin V2-large and ConvNeXtV2-large, require 197 M and 198 M parameters, respectively. They also exhibit substantially longer inference times of 11.64 ms and 9.78 ms, respectively. This highlights the practical advantage of the distilled student model for deployment in resource-constrained clinical settings.

## 5. Discussion

The manual analysis of sperm morphology, a cornerstone in male infertility diagnosis, is fraught with subjectivity and is labor-intensive. While deep learning offers a path to automation, the computational burden of large models often hinders their practical deployment in clinical settings with limited resources. This study investigated the efficacy of a multi-teacher knowledge distillation approach, specifically soft distillation, to develop a lightweight yet accurate student model (SwinV2-base) for automated sperm morphology classification. Instead of relying on a single source, the student leveraged aggregated knowledge from an ensemble of high-capacity teacher models—specifically pairing SwinV2-large with ConvNeXtV2-large or EfficientNetV2-m—to capture diverse feature representations from the challenging, unbalanced Hi-LabSpermMorpho datasets. Our findings demonstrate that the proposed approach, incorporating data augmentation and regularization techniques like stochastic depth and label smoothing, yields notable improvements in performance metrics compared to baseline models. And fine-tuning the teacher model with similar datasets furthers these improvements by transferring valuable domain knowledge to the student model.

The primary contribution of this work lies in showcasing a practical method to overcome the dual challenges of dataset limitations (small size, significant class imbalance) and the deployment constraints of complex deep learning models in a specialized medical imaging domain. For the BesLab dataset, the student model of the single teacher experiment, when trained with both data augmentation and soft distillation, achieved an accuracy of 0.7082 and an weighted F1-score of 0.7033 as in Table 5. This represents an improvement over the baseline model trained without these techniques (Accuracy: 0.6929, Weighted F1-score: 0.6886) and the model trained with augmentation alone (Accuracy: 0.7002, Weighted F1-score: 0.6958). This underscores the synergistic effect of data augmentation in expanding the training data diversity and knowledge distillation in transferring valuable learned representations from the single teacher model.

Data augmentation played a crucial role in mitigating overfitting, a common issue when training deep models on limited datasets like BesLab. By artificially increasing the dataset size six-fold (the original image plus five augmented versions) through various transformations, we provided the model with a richer set of examples, enhancing its ability to generalize. The improvement from the baseline to the augmentation-only model supports this. However, the subsequent improvement with the addition of soft distillation highlights the added value of the teacher-student paradigm. The teacher model, pre-trained on extensive datasets (ImageNet-21k), acts as a regularizer and guides the student model towards a better generalization landscape, even when the teacher’s original training domain differs from the sperm morphology task. The use of a distillation temperature (τ = 8) helped in softening the teacher’s probability distributions, facilitating a smoother knowledge transfer, which is particularly important given the domain mismatch.

The incorporation of stochastic depth (0.3) and label smoothing (0.1) further incrementally improved performance for the BesLab dataset as given in Table 5. Stochastic depth acts as a form of model regularization by randomly dropping layers during training, encouraging the network to learn more robust features. Label smoothing helps in preventing the model from becoming overconfident in its predictions, which can be beneficial, especially with noisy or imbalanced labels inherent in medical datasets.

Furthermore, the experiment where the teacher model was also trained on the Histoplus and GBL datasets showed the highest performance metrics as in Table 5, achieving an accuracy of 0.7082 and an weighted F1-score of 0.7033, indicating that a teacher with more relevant domain knowledge can further enhance the student’s performance.

Our results align with existing literature that advocates for knowledge distillation as a potent technique for model compression and performance enhancement in image classification [18,19]. Specifically, in the context of medical imaging where annotated data can be scarce and expensive to obtain, distillation offers a pathway to leverage large, pre-trained models without incurring their full deployment cost. While studies like Nabipour et al. [38] and Zhang et al. [14] have explored knowledge distillation and related techniques for sperm morphology, our work specifically details the impact of soft distillation combined with augmentation and other regularization methods on a multi-class sperm morphology dataset with significant imbalance, using state-of-the-art Swin Transformer V2 architectures. The confusion matrices (Figure 4) illustrate the model’s per-class performance, revealing that while overall metrics improved, challenges remain for minority classes, a typical issue in imbalanced classification tasks. For instance, classes like ’Long Tail’, which have a very limited number of 42 images, are inherently harder to classify correctly.

To assess the generalizability of our approach, we performed additional experiments on the Histoplus and GBL datasets. We evaluated the impact of domain-enriched supervision through the ’Teacher Train’ setting. In this phase, we implemented a ’leave-one-out’ cross-dataset protocol: for each target dataset used to train the student, the teacher model was fine-tuned on the aggregation of the remaining two complementary subsets (Table 7). The results demonstrated the effectiveness of this strategy across both datasets. For Histoplus, the model achieved an accuracy of 0.7348 and a weighted F1-score of 0.7302. Likewise, on the GBL dataset, this configuration yielded an accuracy of 0.7163, confirming the robustness of the student model when guided by a domain-adapted teacher.

To further enhance the robustness of the student model, we extended our investigation to a multi-teacher knowledge distillation framework. We hypothesized that a student model could benefit from the aggregated knowledge of multiple teacher architectures, as different models often capture distinct feature representations from the same input images. As detailed in Table 7, we experimented with two multi-teacher configurations: combining the primary SwinV2-large teacher with EfficientNetV2-m, and combining SwinV2-large with ConvNeXtV2-large.

The experimental results support the efficacy of this approach. The multi-teacher strategy using SwinV2-large and ConvNeXtV2-large yielded the highest performance across the majority of metrics on the BesLab dataset, achieving an accuracy of 0.7094 and an weighted F1-score of 0.7052. This represents a slight but consistent improvement over the single-teacher approach (Accuracy: 0.7082). This performance advantage was also observed when testing on the auxiliary datasets; for instance, the SwinV2/EfficientNet combination achieved the highest reported accuracy of 0.7361 on the Histoplus dataset.

The success of the multi-teacher framework can likely be attributed to the architectural diversity of the teachers. While Swin Transformers excel at capturing long-range dependencies via self-attention mechanisms, Convolutional Neural Networks (like EfficientNet and ConvNeXt) possess strong inductive biases for local feature extraction. By distilling the averaged logits from both architecture types, the student model (SwinV2-base) learns a more generalized representation that is less susceptible to the specific biases or errors of a single teacher architecture. This “ensemble-like” guidance effectively smooths the optimization landscape for the student, resulting in a model that is more robust to the high variability inherent in sperm morphology images.

The granular analysis of difference matrices (Figure 5) reveals that the Multi-Teacher framework does not merely provide a uniform performance boost, but rather induces a fundamental shift in the model’s decision boundaries. A prominent effect observed in the BesLab dataset is the trade-off between the majority ‘Amorphous Head’ (Class 1) and the minority ‘Narrow Acrosome’ (Class 3). While the ensemble effectively reduced false positives for the majority class—correcting instances where Amorphous heads were mistaken for Narrow Acrosomes—it simultaneously adopted a more conservative approach, absorbing actual Narrow Acrosome samples into the majority category. Despite this specific sensitivity loss, the Multi-Teacher architecture demonstrated superior feature extraction capabilities for distinct anomalies; specifically, ‘Vacuolated Head’ (Class 8) and ‘Asymmetric Neck’ (Class 9) showed consistent and significant performance gains across all three staining protocols. This suggests that the combination of Swin Transformer and ConvNeXt features is particularly robust in capturing internal textural defects and geometric asymmetries. However, the systematic deterioration observed for ‘Thick Neck’ (Class 10) across all datasets indicates a limitation: the ensemble averaging process appears to soften the sharp decision boundaries required to define neck thickness, suggesting that specific morphological traits may still benefit from the sharper, albeit potentially noisier, inductive biases of a single-teacher constraint.

Table 11 benchmarks the proposed Multi-Teacher framework against the current state-of-the-art methods applied to the Hi-LabSpermMorpho dataset. The comparison includes the baseline EfficientNetV2-M [23], and the Category-aware Splitter Model [36], all of which utilized complex architectural designs to address the 18-class classification challenge. The experimental results demonstrate that the proposed distillation method consistently outperforms these existing approaches across all three staining techniques. On the BesLab dataset, our method achieved an accuracy of 70.94%, surpassing the previously best-performing Splitter Model (69.43%). This superiority is even more pronounced in the Histoplus and GBL datasets, where accuracies reached 73.61% and 71.63%, respectively. Notably, the performance gap is widest in the GBL dataset, where the proposed framework exceeds the closest competitor by approximately 3.1 percentage points (71.63% vs. 68.41%). This indicates that transferring aggregated knowledge from complementary teachers (SwinV2 and ConvNeXtV2) provides a more robust generalization strategy than the purely architectural ensemble or task-splitting methods employed in prior studies. By effectively distilling both global and local feature representations, our student model captures subtle morphological nuances that standard training paradigms or divide-and-conquer strategies might miss.

Furthermore, to explicitly validate the contribution of the knowledge distillation process, our framework was rigorously evaluated against strong baselines without distillation. Internally, the distilled model significantly outperformed the identically configured standalone student model (Table 5, Exp. 2). Externally, as benchmarked in Table 11, our multi-teacher distillation approach surpassed recent high-capacity, non-distilled state-of-the-art models (such as splitter networks), proving that the aggregated teacher supervision provides superior regularization and feature extraction compared to training even strong standalone architectures directly on the imbalanced dataset. However, we acknowledge that while our regularization strategy prevents total minority class collapse, it does not fully resolve the low sensitivity observed in extreme minority classes (e.g., ‘Long Tail’ and ‘Asymmetric Neck’). The extreme scarcity of training samples for these specific defects means that architectural regularizers alone are insufficient to achieve high F1-scores without fundamentally augmenting the feature space.

### Future Work

Despite the promising results, this study has limitations. Our exploration focused on a particular type of knowledge distillation (soft distillation). Future work could explore other distillation methods (e.g., feature-based, relation-based) or different teacher-student pairs, potentially including even more compact student models for edge deployment. Crucially, to address the persistently low F1-scores in extreme minority classes, future studies must move beyond architectural regularization. Integrating dedicated scarcity-handling techniques—such as few-shot learning paradigms or synthetic data generation via diffusion models—in conjunction with our distillation framework is necessary to fundamentally balance the feature representation of underrepresented sperm abnormalities [44]. The selection of hyperparameters for distillation (temperature, distillation weight) was based on experimentation; more systematic optimization approaches could be explored. Additionally, while this study utilized simple arithmetic averaging for multi-teacher logit aggregation to maximize regularization, future studies should investigate performance-based or dynamic weighting mechanisms to potentially extract even more nuanced supervision signals. Furthermore, since our interpretations regarding the impact of specific staining protocols were based on observational data across independent patient cohorts, future research should incorporate controlled experiments. Digitizing identical sperm samples across multiple staining techniques would quantitatively isolate and validate the causal impact of stains on deep learning feature extraction. Finally, prospective clinical validation studies are crucial to translate these automated systems into tools that can reliably assist embryologists and andrologists in routine practice.

In conclusion, this study demonstrates that a knowledge distillation approach, augmented with appropriate data pre-processing and regularization, can effectively train smaller, more efficient deep learning models for sperm morphology classification with gains in accuracy. This offers a viable strategy for developing deployable automated systems in resource-constrained clinical environments, potentially improving the efficiency and objectivity of male infertility diagnosis.

## Figures and Tables

**Table 1:** Comparison of Knowledge Distillation Approaches in Sperm Morphology Classification.

Study	Distillation Strategy	Dataset(s)	Classification Scope	Limitations & Addressed Gaps
Nabipour et al. [38]	Less-supervised learning (Anomaly detection via normal samples)	MHSMA	Binary (Normal vs. Abnormal parts)	Focuses solely on anomaly detection rather than multi-class defect categorization. Uses a single dataset domain.
Zhang et al. [14]	Unsupervised anatomical feature distillation (pseudo-masks)	SCIAN, HuSHeM	Multi-class (Head only)	Limited to sperm head morphology. Relies on single-teacher distillation, prone to single-domain architectural biases.
**Proposed Method**	**Multi-Teacher Soft Distillation with Cross-Dataset Training**	**Hi-LabSpermMorpho (BesLab, Histoplus, GBL)**	**18 fine-grained classes (Head, Neck, Tail)**	**Addresses extreme class imbalance and cross-domain stain variations by aggregating diverse architectural features (SwinV2 + ConvNeXtV2).**

**Table 2:** Image counts for each class across the BesLab, Histoplus, and GBL datasets.

Morphology Class	BesLab	Histoplus	GBL
Amorf Head	3572	2861	1537
Double Head	48	35	52
Narrow Acrosome	2055	1992	1355
Pin Head	782	423	118
Pyriform Head	979	1843	1123
Round Head	257	415	201
Tapered Head	1399	1356	1150
Vacuolated Head	1697	604	509
Asymmetric Neck	366	492	458
Thick Neck	1978	1340	934
Thin Neck	192	173	180
Twisted Neck	1154	1566	855
Curly Tail	1445	1385	955
Double Tail	200	183	81
Long Tail	42	128	106
Short Tail	991	538	359
Twisted Tail	706	2502	2277
Normal	599	433	381

**Table 3:** Data augmentation pipelines applied to the training set. All strategies conclude with a central crop to a final resolution of 256×256 pixels. Prior to these operations, images are padded to prevent artifacts.

Strategy	Padding	Primary Transform	Secondary Transform
1	Edge (50 px)	Rotation ($\pm 30^{{}^{\circ}}$)	—
2	Edge (30 px)	Height Shift (0–0.1)	Shear ($10^{{}^{\circ}}$)
3	Edge (30 px)	Horizontal Flip (p=0.5)	Zoom (0.9–1.0)
4	Reflect (20 px)	Width Shift (0–0.1)	Height Shift (0–0.1)
5	Reflect (20 px)	Horizontal Flip (p=0.5)	Height Shift (0–0.1)

**Table 4:** Training parameters for Student and Teacher models.

Parameters	Student Model	Teacher Model
Optimizer	AdamW	AdamW
Learning Rate	0.00002	0.00002
Learning Rate Scheduler	StepLR	StepLR
LR Scheduler Gamma	0.45	0.5
LR Scheduler Step Size	1	2
Weight Decay	0.0001	0.01
Epochs	10	4
Batch Size	4	4
Stochastic Depth	0.3	-
Label Smoothing	0.1	-
Distillation Weight	0.3	-
Distillation Temperature	8	-

**Table 5:** Summary of results for the single teacher experiments with different settings applied on BesLab dataset (Wei. is the abbreviation for Weighted).

Exp.	Aug	Dist	SD	LS	TT	Accuracy	Wei. F1	Wei. Precision	Wei. Recall	Macro F1
1	✘	✘	✘	✘	✘	0.6929 ± 0.0050	0.6886	0.6865	0.6929	0.6433 ± 0.0169
2	✔	✘	✘	✘	✘	0.7002 ± 0.0037	0.6958	0.6945	0.7002	0.6587 ± 0.0081
3	✔	✔	✘	✘	✘	0.7063 ± 0.0039	0.6993	0.6984	0.7063	0.6444 ± 0.0143
4	✔	✔	✔	✘	✘	0.7063 ± 0.0053	0.6996	0.6978	0.7063	0.6382 ± 0.0153
5	✔	✔	✘	✔	✘	0.7067 ± 0.0046	0.6998	0.6993	0.7067	0.6456 ± 0.0119
6	✔	✔	✔	✔	✘	0.7072 ± 0.0047	0.7004	0.6988	0.7072	0.6413 ± 0.0187
7	✔	✔	✔	✔	✔	0.7082 ± 0.0064	0.7033	0.7033	0.7082	0.6624 ± 0.0102

Note: Exp.: Experiment Setting; Aug: Data Augmentation; Dist: Knowledge Distillation; SD: Stochastic Depth; LS: Label Smoothing; TT: Teacher Train; ✔: Applied; ✘: Not Applied.

**Table 6:** Statistical significance (paired *t*-test) of the single teacher distillation improvements across 5-fold cross-validation on the BesLab dataset.

Comparison	Metric	t-Value	*p*-Value	Significance (p<0.05)
Exp. 7 vs. Exp. 1	Macro F1	3.1830	0.0334	Yes
Exp. 7 vs. Exp. 2	Macro F1	0.8931	0.4223	No
Exp. 7 vs. Exp. 3	Macro F1	4.2303	0.0134	Yes
Exp. 7 vs. Exp. 4	Macro F1	3.9221	0.0172	Yes
Exp. 7 vs. Exp. 5	Macro F1	2.2154	0.0911	No
Exp. 7 vs. Exp. 6	Macro F1	3.1358	0.0350	Yes

**Table 7:** Comparison of Single and Multi-Teacher Training performances on different datasets.

Method	Model Configuration	Dataset	Accuracy	Wei. F1	Wei. Precision	Wei. Recall	Macro F1
Single Teacher	SwinV2-large	BesLab	0.7082 ± 0.0064	0.7033	0.7033	0.7082	0.6624 ± 0.0102
		Histoplus	0.7348 ± 0.0057	0.7302	0.7312	0.7348	0.6818 ± 0.0120
		GBL	0.7163 ± 0.0073	0.7095	0.7115	0.7163	0.6644 ± 0.0113
Multi Teacher	SwinV2-large + EfficientNetV2-m	BesLab	0.7088 ± 0.0067	0.7040	0.7036	0.7088	0.6666 ± 0.0101
		Histoplus	0.7344 ± 0.0059	0.7313	0.7318	0.7344	0.6881 ± 0.0154
		GBL	0.7129 ± 0.0042	0.7073	0.7076	0.7129	0.6630 ± 0.0133
	SwinV2-large + ConvNeXtV2-large	BesLab	0.7094 ± 0.0068	0.7052	0.7053	0.7094	0.6765 ± 0.0056
		Histoplus	0.7361 ± 0.0047	0.7324	0.7327	0.7361	0.6895 ± 0.0093
		GBL	0.7153 ± 0.0043	0.7107	0.7114	0.7153	0.6726 ± 0.0065

**Table 8:** Statistical significance (paired *t*-test) of the Multi-Teacher improvements in Macro F1 across 5-fold cross-validation.

Dataset	Comparison (Multi vs. Single)	t-Value	*p*-Value	Significance (p<0.05)
BesLab	SwinV2 + ConvNeXtV2-large vs. SwinV2-large	4.9327	0.0079	Yes
Histoplus	SwinV2 + ConvNeXtV2-large vs. SwinV2-large	2.8506	0.0464	Yes
GBL	SwinV2 + ConvNeXtV2-large vs. SwinV2-large	3.2177	0.0324	Yes

**Table 9:** The detailed class-based performance measurement for all datasets.

Classes	BesLab				Histoplus				GBL			
	F1	Prec.	Rec.	PR-AUC	F1	Prec.	Rec.	PR-AUC	F1	Prec.	Rec.	PR-AUC
Amorf Head	0.6345	0.6400	0.6291	0.6926	0.6457	0.6532	0.6383	0.7055	0.6199	0.6050	0.6357	0.6626
Asymmetric Neck	0.2476	0.4088	0.1776	0.2337	0.3140	0.4474	0.2419	0.3437	0.3295	0.4715	0.2533	0.3489
Curly Tail	0.9003	0.8812	0.9203	0.9617	0.8806	0.8520	0.9111	0.9449	0.8250	0.7834	0.8712	0.8983
Double Head	0.7750	0.9687	0.6458	0.7420	0.6349	0.7143	0.5714	0.7699	0.8132	0.9487	0.7115	0.9191
Double Tail	0.8763	0.9477	0.8150	0.9044	0.8626	0.8674	0.8579	0.9360	0.7792	0.8219	0.7407	0.8396
Long Tail	0.3390	0.5882	0.2381	0.4534	0.3676	0.5965	0.2656	0.4562	0.1935	0.6667	0.1132	0.3355
Narrow Acrosome	0.6706	0.6480	0.6947	0.7390	0.6967	0.6737	0.7214	0.7716	0.6832	0.6837	0.6827	0.7567
Normal	0.8545	0.8647	0.8445	0.9250	0.7173	0.7258	0.7090	0.7968	0.7378	0.8153	0.6737	0.8192
Pin Head	0.9910	0.9961	0.9859	0.9915	0.9905	0.9976	0.9835	0.9928	0.9573	0.9655	0.9492	0.9762
Pyriform Head	0.6459	0.6175	0.6769	0.6924	0.7461	0.7363	0.7562	0.8210	0.7317	0.6991	0.7676	0.8120
Round Head	0.5500	0.6612	0.4708	0.6284	0.6192	0.6316	0.6072	0.6833	0.5514	0.6036	0.5075	0.6552
Short Tail	0.8757	0.8735	0.8779	0.9382	0.8542	0.8707	0.8383	0.9118	0.7588	0.7686	0.7493	0.8090
Tapered Head	0.6846	0.6687	0.7012	0.7658	0.6873	0.6873	0.6873	0.7599	0.6791	0.6782	0.6800	0.7535
Thick Neck	0.6582	0.6619	0.6545	0.7358	0.6344	0.6023	0.6701	0.6934	0.6199	0.6115	0.6285	0.6451
Thin Neck	0.3211	0.4486	0.2500	0.2926	0.4364	0.5882	0.3468	0.4375	0.4158	0.5859	0.3222	0.4831
Twisted Neck	0.7975	0.7725	0.8241	0.8768	0.8276	0.8290	0.8263	0.9082	0.7760	0.7265	0.8327	0.8612
Twisted Tail	0.7211	0.6732	0.7762	0.8230	0.8782	0.8776	0.8782	0.9413	0.8851	0.8758	0.8946	0.9445
Vacuolated Head	0.6350	0.6396	0.6305	0.7007	0.6177	0.5906	0.6177	0.6558	0.6021	0.5935	0.6110	0.6210
Accuracy	0.7094				0.7361				0.7163			
Macro Avg.	0.6765	0.7200	0.6563	0.7276	0.6895	0.7190	0.6755	0.7516	0.6644	0.7169	0.6458	0.7300
Weighted Avg.	0.7052	0.7053	0.7094	0.7673	0.7324	0.7327	0.7361	0.7965	0.7095	0.7115	0.7063	0.7706

**Table 10:** Comparison of number of parameters and inference time of models per image.

Role	Model Architecture	Parameters (M)	Inference Time per Image (ms)
Teacher 1	SwinV2-large	197	11.64
Teacher 2	ConvNeXtV2-large	198	9.78
Student	SwinV2-base	88	6.64

**Table 11:** Sperm morphology analysis performance comparison of the proposed and previously applied methods for the Hi-LabSpermMorpho dataset (Wei. is abbreviation for Weighted; bold formatting indicates the proposed method and the best performance values achieved for each metric).

Staining Technique	Class Number	Model	Accuracy	Wei. F1	Wei. Precision	Wei. Recall
BesLab	18	EfficientNetV2-M [23]	65.05	64.39	64.35	65.05
		Splitter Model + Ensemble [36]	69.43	68.77	68.84	69.43
		**Proposed Multi-Teacher Method**	**70.94**	**70.52**	**70.53**	**70.94**
Histoplus	18	EfficientNetV2-M [23]	67.42	67.13	67.00	67.42
		Splitter Model + Ensemble [36]	71.34	70.60	70.67	71.34
		**Proposed Multi-Teacher Method**	**73.61**	**73.24**	**73.27**	**73.61**
GBL	18	EfficientNetV2-M [23]	63.58	63.10	63.03	63.46
		Splitter Model + Ensemble [36]	68.41	67.54	67.73	68.41
		**Proposed Multi-Teacher Method**	**71.63**	**70.95**	**71.15**	**71.63**

## Data Availability

The datasets utilized in this work namely Hi-LabSpermMorpho can be found in online repositories. Repository names and accession numbers: https://github.com/Yildiz-Hi-Lab/Hi-LabSpermMorpho (accessed on 16 April 2026).
